# Functional optoacoustic neuro-tomography for scalable whole-brain monitoring of calcium indicators

**DOI:** 10.1038/lsa.2016.201

**Published:** 2016-12-02

**Authors:** X Luís Deán-Ben, Gali Sela, Antonella Lauri, Moritz Kneipp, Vasilis Ntziachristos, Gil G Westmeyer, Shy Shoham, Daniel Razansky

**Affiliations:** 1 Institute for Biological and Medical Imaging (IBMI), Helmholtz Center Munich, Neuherberg, Germany; 2 Institute of Developmental Genetics, Helmholtz Center Munich, Neuherberg, Germany; 3 Department of Medicine, Technical University of Munich, Munich, Germany; 4 Department of Biomedical Engineering, Technion-Israel Institute of Technology, Haifa, Israel

**Keywords:** functional neuro-imaging, genetically encoded calcium indicators, high spatiotemporal resolution, large-scale brain activity, optoacoustic tomography, photoacoustics, real-time imaging

## Abstract

Non-invasive observation of spatiotemporal activity of large neural populations distributed over entire brains is a longstanding goal of neuroscience. We developed a volumetric multispectral optoacoustic tomography platform for imaging neural activation deep in scattering brains. It can record 100 volumetric frames per second across scalable fields of view ranging between 50 and 1000 mm^3^ with respective spatial resolution of 35–200 μm. Experiments performed in immobilized and freely swimming larvae and in adult zebrafish brains expressing the genetically encoded calcium indicator GCaMP5G demonstrate, for the first time, the fundamental ability to directly track neural dynamics using optoacoustics while overcoming the longstanding penetration barrier of optical imaging in scattering brains. The newly developed platform thus offers unprecedented capabilities for functional whole-brain observations of fast calcium dynamics; in combination with optoacoustics' well-established capacity for resolving vascular hemodynamics, it could open new vistas in the study of neural activity and neurovascular coupling in health and disease.

## Introduction

Neuronal activation occurs concurrently or in a highly coordinated manner in different areas across the nervous system, reflecting functional interconnection between specialized neuronal subcircuits. Imaging neuronal activation with high temporal and spatial resolution over an entire intact brain, including deep and normally inaccessible areas, could thus play a critical role in the attempt to decipher the fundamental operating principles underlying neural circuit activity. Major efforts are underway to advance the ability to optically image the activity of large, distributed neural populations^1, 2^. Recently, these efforts have led to whole-brain activity imaging in transparent organisms using single^3^ and multiphoton^4^ light-sheet and light-field^5^ microscopy. However, these approaches are unable to tackle deep neural activity in intact scattering brains where current state-of-the-art optical imaging strategies based on rapidly scanning multiphoton microscopy are limited to volumes below 1 mm^3^ because of limited penetration depth and field of view (FOV).

Conversely, optoacoustic imaging, an extremely powerful approach with demonstrated capacity for centimeter-scale penetration into highly scattering tissues^6^, can potentially provide functional neural imaging beyond the limit of current optical imaging technologies. To date, functional optoacoustic brain imaging mainly focused on probing hemodynamics and blood oxygenation variations^7, 8, 9^, slow and delayed processes that only indirectly reflect neural activation. In contrast, modern approaches toward imaging neural activation are largely based on measuring calcium dynamics, which more directly correlate with neural activity. Calcium dynamics are conventionally tracked using fluorescent sensors, which change their fluorescence output because of a change in their extinction coefficient, fluorescence quantum yield or both as a function of intracellular calcium concentrations^10, 11^. Recent advances in the field of genetically encoded calcium indicators (GECIs) have provided a variety of genetically modified reporter animal models with calcium indicators in specific neuronal structures^12, 13, 14^, which in some cases can give rise to signals down to the level of single action potentials^13, 15^. The new generation of GECIs has several key advantages over organic indicators—they can be expressed over very large volumes and entire brains using the generation of transgenic animals or viral expression; they allow repeated imaging in the same region and animal over multiple days and even up to years; they can be targeted for expression to specific cell populations and even in specific cellular organelles, allowing for flexible, diverse and precise associated methodologies not nearly possible with the traditional organic staining methods.

We hypothesized that some members of the GCaMP family of fluorescent GECIs could provide a suitable calcium-dependent optoacoustic contrast because of their strong calcium-dependent extinction changes^12^. To test this hypothesis, we selected the zebrafish reporter line HuC:GCAMP5G that exhibits expression of the calcium sensor in a large fraction of neurons to obtain high-resolution optoacoustic measurements from larvae and isolated adult brains.

## Materials and methods

### The FONT set-up

**Figure 1:**
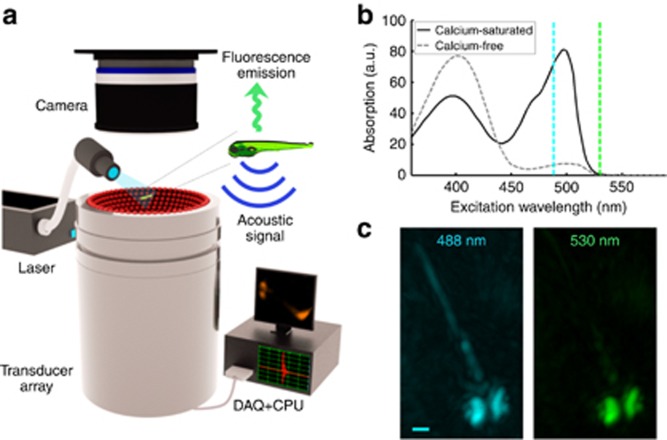
FONT set-up. (**a**) The imaged sample is placed in the vicinity of the geometrical center of a custom-made spherical ultrasound detection array. Light illumination of the entire object is provided *via* an optical fiber bundle. The generated optoacoustic signals are acquired by the array transducer, whereas the induced fluorescence is simultaneously recorded by the high-speed sCMOS camera. (**b**) Spectral dependence of the absorbance on calcium-bound versus calcium-free GCaMP5G (reproduced from Ref. 12). (**c**) Optoacoustic images of a 5-day-old HuC:GCAMP5G larva acquired at 488 and 530 nm (maximal intensity projection views through volumetric reconstructions are shown). Whereas the pigmented eyes are strongly absorbing at both wavelengths, GCaMP5G is hardly absorbing at 530 nm; thus, neural tissue is much more visible at 488 nm (scale bar=250 μm).

To enable tomographic imaging of whole scattering brains of different sizes, we developed a new generation of functional optoacoustic neuro-tomography (FONT) platform that can simultaneously monitor calcium activity in all five dimensions, i.e., space, time and wavelength (spectrum), with a scalable spatiotemporal resolution performance. As shown in Figure 1a, optoacoustic responses are generated by a tunable optical parametric oscillator laser source (InnoLas Laser GmbH, Krailling, Germany) that broadly illuminates the imaged sample via custom-made silica fused-end fiber bundle (CeramOptics GmbH, Bonn, Germany). The light fluence at the sample was measured at 3 mJ cm^−2^. The maximal volumetric imaging frame rate of the system is determined by the 100 Hz pulse repetition rate of the laser. The optoacoustic detection system was designed to render real-time volumetric (three-dimensional (3D)) tomographic capacity with unprecedented spatiotemporal resolutions at depths beyond penetration limits of modern optical microscopes (~0.5 mm in highly scattering brains^16^). In general, spatial resolution in optoacoustic tomography scales with the available bandwidth of the ultrasound detection system, which, as opposed to light microscopy, is not affected by light scattering in tissues. To enable scalability in imaging brains of different sizes, three different implementations of custom-built piezocomposite matrix arrays (termed FONT3, FONT2 and FONT1) are considered herein. The systems offer real-time imaging with scalable FOV of 50–1000 mm^3^ and respective spatial resolution varying between 35 and 200 μm. Under FONT1 we refer to the previously reported optoacoustic imaging system^17^, employing 256-element spherical ultrasound array with 4 MHz central frequency and 90^o^ solid angular coverage, which was shown to enable imaging with 200 μm resolution over a 1000 mm^3^ volumetric FOV. Higher spatial resolution is enabled herein with the newly developed FONT2 and FONT3 systems consisting of 512-element spherical matrix arrays (Supplementary Fig. S1). Their individual elements have an approximate diameter of 2.5 mm (FONT2) and 1 mm (FONT3) and frequency response as plotted in Supplementary Fig. S1d. The radii of the spherically shaped arrays are 30 mm (FONT2) and 15 mm (FONT3) and the active detection surfaces cover respective solid angles of ~1.3*π* (140^o^) and ~2*π* (180^o^), which allows for an excellent tomographic coverage and high detection sensitivity within the effective FOV of each system. This major performance improvement over previous state-of-the-art implementations^17^ enables efficient imaging of brains at different scales, from the larval zebrafish nervous system with volumes below 1 mm^3^ up to the whole adult mouse brain occupying ~420 mm^3^. The space between the active transducer surface and the imaged samples was filled with an agar gel medium to guarantee acoustic coupling and convenient placement of the samples. The parallel digital acquisition (DAQ) platform was further custom-developed to enable simultaneous sampling of the 512 ultrasound channels and online data transmission over 1 Gb Ethernet connection to the host PC equipped with a high-end graphic processing unit (HD7900, AMD Radeon) for real-time data processing. The developed digital acquisitions operate at sampling frequencies of 40 megasamples per second (FONT2) and 125 megasamples per second (FONT3).

### Image reconstruction methods

Real-time volumetric optoacoustic image rendering and pre-view during the experiment was realized with a GPU-accelerated implementation of standard filtered back-projection reconstruction^18^. The collected data were further processed off-line with a self-developed accurate 3D model-based reconstruction algorithm^19^ for better quantitative assessment of dynamic absorption contrast and calcium concentration changes. This reconstruction procedure is based on numerically solving an inversion problem corresponding to the minimization of the least square difference between the measured signals and those predicted by a discretized version of the forward optoacoustic model. Model-based reconstruction further enables accounting for the effect of the finite aperture of individual detection elements^20^, which leads to contrast enhancement and better accuracy of the reconstructed images. A uniform non-attenuating acoustic medium was assumed for the image reconstruction since acoustic attenuation in agar is insignificant while its acoustic impedance is further very similar to that of water and soft tissues. The value of the speed of sound used for the reconstruction was determined during calibration experiments with microspheres and further fine-tuned for best image appearance of the zebrafish specimens.

### Concurrent epifluorescence image acquisition

Systematic comparison of optoacoustic and fluorescence contrasts was enabled by integrating the optoacoustic tomographic system with a rapid high-resolution sCMOS camera (model: pco.edge 4.2, PCO AG, Kelheim, Germany), synchronized with the pulsed-laser source. The illumination was altered for this purpose and directed at the sample laterally to avoid blinding the camera. As a result, large optically opaque samples (like the adult brain) were not always uniformly illuminated. The fluorescence emission was picked up by the camera using a longpass filter (cutoff wavelength 500 nm). In order to avoid damage to the camera, simultaneous optoacoustic and fluorescent imaging was performed with a single-excitation wavelength of 488 nm. The interrogation laser allows for fast wavelength tuning on a per-pulse basis, thus the system also effectively enables fast acquisition of five-dimensional tomographic data sets, namely, time-resolved volumetric data at multiple wavelengths. The multispectral imaging frame rates are then accordingly reduced by the number of acquired wavelengths. Yet, our imaging rates in all experiments were significantly higher than the characteristic response times of the calcium indicators^12^.

### Animal maintenance

Zebrafish maintenance and breeding were conducted under standard conditions^21^. The fertilized eggs from a HuC:GCaMP5G, originally generated by the laboratory of Michael B. Orger and obtained as a kind gift from the laboratory of Hernán López-Schier, were raised in embryo medium at 28 °C under standard light/dark cycle. All procedures involving animals and their care were conducted in conformity with the institutional guidelines and with approval from the Government of Upper Bavaria.

### Zebrafish larvae preparation and stimulation

For the experiments with freely swimming larvae, the latter was released into a small (6 × 6 × 3 mm^3^) chamber, placed at the focal area of the optoacoustic matrix array detector and filled with fish water. The animals were given 30–60 s to acclimate before starting the stimulation. During the experiments with immobilized fish, six-day-old HuC:GCaMP5G larvae were placed in fish water containing 0.5% of muscle relaxant (Gallamine triethiodide, Sigma-Aldrich, Munich, Germany) briefly before they were embedded in 0.5% low-melting-temperature agarose and positioned into the FOV of the optoacoustic detector. To elicit calcium transients, 50 mM Pentylenetetrazole (PTZ)^22^ was applied via a syringe pump (flow rate of 5 μL s^−1^, volume 100–200 μL), directly into the surrounding of the larvae containing ~200 μL of fish water.

### Brain isolation and mounting

Adult HuC:GCaMP5G zebrafish (1.5 months old) were killed. Their brains were isolated following a standard procedure^23^ and quickly immersed in artificial fish cerebral spinal fluid^24^. The brains were then mounted in 0.5% low-melting-temperature agarose dissolved in artificial fish cerebral spinal fluid and positioned within the imaged FOV. PTZ was then similarly applied in the vicinity of the brain.

## Results and discussion

The resolution and FOV of the systems were characterized by imaging absorbing polyethylene microspheres (Cospheric BKPMS, size 45–53 μm for FONT2 and Cospheric BKPMS, size 20–27 μm for the FONT3 characterization). The particles were embedded in an agar matrix mimicking optical scattering in tissues. The resolution was estimated from the full width at half maximum (FWHM) of the reconstructed absorption profiles for the microspheres located approximately in the center of the detection array (Supplementary Fig. S1c). In order to account for the size of the microspheres, it was assumed that the measured FWHM corresponds to the convolution of two Gaussian functions representing the actual absorption profile of the sphere and the point spread function of the system. In this way, the resolution was estimated as the mean square difference between the measured FWHM and the diameter of the microsphere. The FOV was estimated by imaging a sparse distribution of microspheres. Specifically, it was determined as the size of a region enclosing particles that generate optoacoustic response higher than 50% of the maximum signal in the images (Supplementary Fig. S1b). Note that both the combined sensitivity and spatial resolution performance of the spherical array deteriorates away from the center of the spherical tomographic detection geometry. The effective FOV further depends on the frequency content of the emitted optoacoustic signals, such that larger absorbing structures mainly emitting lower frequencies can be efficiently imaged over a larger volume.

To explore the fundamental spectral signature of our experimental system, 5-day-old HuC:GCaMP5G zebrafish larvae were imaged using two distinct wavelengths: 488 nm peak absorption wavelength of GCaMP5G and 530 nm where GCaMP5G absorption is negligible (Figure 1b). While the eyes of the larva, with their characteristically highly absorbing melanin pigmentation, are readily visible in both optoacoustic images, the tissues expressing GCaMP5G do not yield a strong signal at 530 nm but provide clear contrast at 488 nm, where the protein is highly absorbing (Figure 1c). Note that the temporal resolution of our systems is high enough to allow tracking of freely moving organisms, for example, freely swimming young larvae, which was imaged using the high-resolution FONT3 system (Supplementary Video 1).

**Figure 2:**
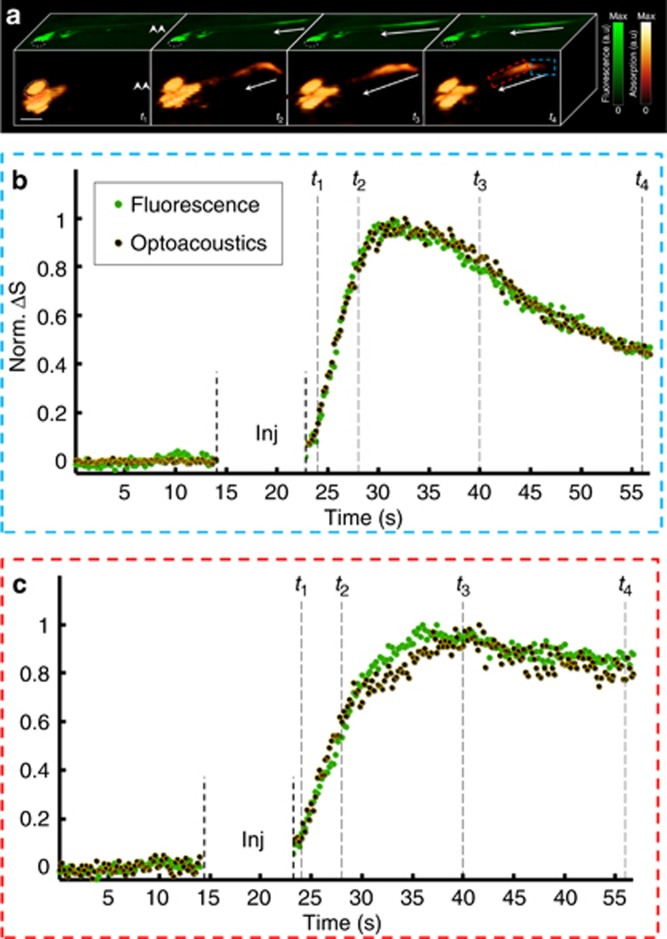
Imaging of neuronal activation in HuC:GCAMP5g zebrafish larvae. (**a**) Planar epifluorescence (green) and 3D optoacoustic (absorption contrast, orange) images of a 6-day-old larvae at four time points after injection of neurostimulant show a similar dynamics of the signals (scale bar=500 μm). The arrows show direction of the activation as it progresses from the posterior (site of injection marked by a double arrow in the left frame) to the anterior part of the tail. Eyes are marked with a dotted line. (**b**) Traces of the fluorescence and optoacoustic signals recorded at 25 frames per second show calcium dynamics in the posterior region of the spinal cord (marked by red rectangle in **a**). (**c**) Corresponding traces in the anterior region (marked by the blue rectangle). The time points shown in **a** are marked by vertical dotted lines. Since both the background fluorescence and optoacoustic signals were close or below the noise level, the changes in the signals (Δ*S*) were normalized to unity instead of being divided by the respective resting signals. The injection phase caused image artifacts and is therefore excluded from the graphs.

First, we tested whether we were able to detect neural activity using a simple and commonly employed preparation of immobilized HuC:GCaMP5G zebrafish larvae (*n*=5). For this purpose, the larvae were exposed to a neuroactivating agent (PTZ) known to induce fast ictal-like spikes in the nervous system that alter their swimming behavior^22^, most likely by interfering with GABAergic signaling^25^. Exposure to PTZ caused robust calcium waves propagating from the posterior (site of drug injection) to the anterior spinal cord region (Figure 2). Strong correlations between the simultaneously acquired optoacoustic and fluorescent signals were observed during these evoked calcium transients both at the posterior-most (*R*^2^=0.98) and in the medial (*R*^2^=0.97) regions, providing confirmation that GCaMP5G calcium sensing can be read out via optoacoustics (Figure 2 and Supplementary Video 2).

**Figure 3:**
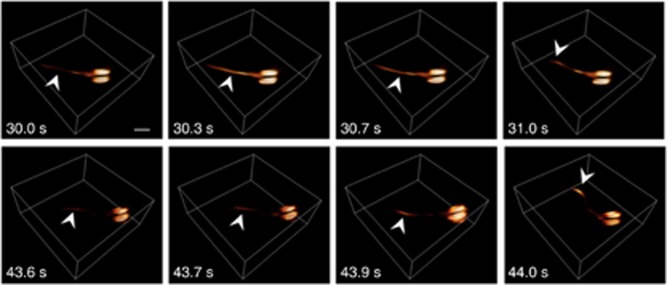
Activation in freely swimming larvae. Two separate activation events, as captured by volumetric optoacoustic tomography, are shown. Following injection of the neurostimulating agent at approximately *t*=0, the larva occasionally stops swimming while experiencing a surge of activation through its tail (the arrows point to the location of optoacoustic signal increase) before it starts moving promptly to a new position (notice movement of the tail in the two rightmost frames). Scale bar=500 μm.

We also examined FONT’s ability to non-invasively measure neural activity during natural behavior. Studying neural processing during unrestrained motion represents an outstanding technical challenge that motivated the recent introduction of a number of experimental paradigms for studying behaving zebrafish larvae using, for example, bioluminescence imaging^26^ as well as light sheet^27^ and light-field^5^ microscopy (note, however, that these strategies cannot provide volumetric information in scattering brains). Here we were able to perform high-speed volumetric imaging of larvae that were allowed to swim freely in an ~0.5-cm^2^-area chamber; following exposure to the neurostimulant, rapid movements followed by long-resting periods were observed. The optoacoustically recorded calcium signal dynamics appear to have characteristic timescales between 0.1 and 0.3 s during this behavior and revealed an increase of up to ΔOA/OA_0_=1.8 in the GCaMP5 signals in the spinal cord just before the animal moves (as indicated by arrows in Figure 3).

**Figure 4:**
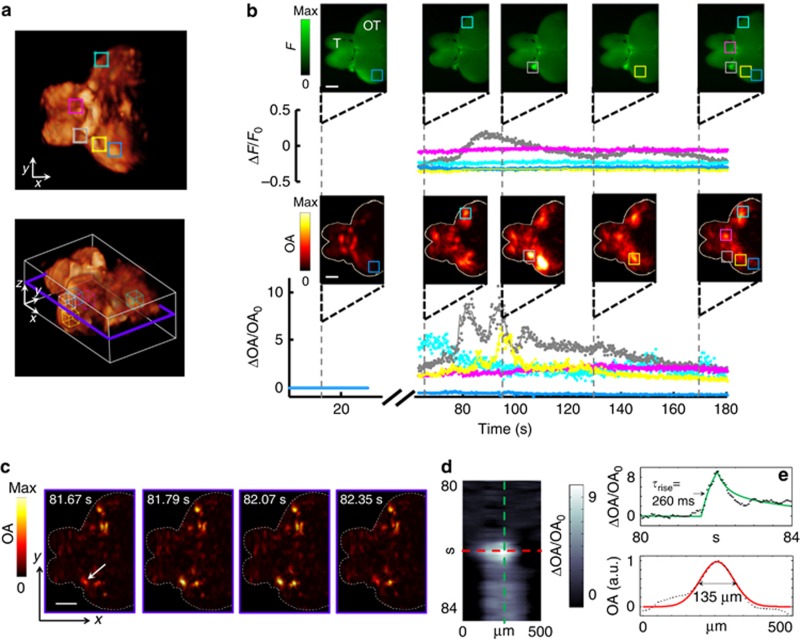
Monitoring activity in isolated scattering adult brains. (**a**) Typical 3D optoacoustic image acquired from a highly scattering brain of an adult fish in its resting state (two side views and one isometric view of the 3D reconstruction are shown). The telencephalon (T) and most of the optic tectum (OT) are clearly visible. Five 0.3 × 0.3 × 0.3 mm^3^ VOIs were chosen at different locations and depths within the brain. (**b**) Traces of the fluorescence (top) and optoacoustic (bottom) signal changes recorded at 25 frames per second are shown for the five regions in corresponding colors (all signal changes are normalized to the resting signal levels). Note that the optoacoustic traces are calculated over volumes, whereas the fluorescent signals are calculated over the roughly corresponding planar areas. Snapshot images (not normalized) acquired at five different time points before and after introduction of the neurostimulant are further shown (scale bar=500 μm). The volumetric optoacoustic images are shown here as maximum intensity projections along the *z* axis. The injection phase caused image artifacts and is therefore excluded from the graphs. (**c**) Time-resolved images from a single (unaveraged) slice through the 3D optoacoustic data. Approximate location of the slice is indicated by a blue frame in **a**. (**d**) Close-up spatiotemporal resolution analysis of a single line (orientation of the line is shown by an arrow in **c**). (**e**) Temporal and spatial profiles through the image in **d**, revealing characteristic optoacoustic signal constants of 260 ms and 135 μm, respectively. Solid green and red lines correspond to fits through the individual data points.

Next, we examined the imaging performance of FONT2 in isolated brains of adult HuC:GCaMP5G zebrafish (*n*=4) having approximate dimensions of 2 × 3 × 4 mm, which were exposed to the neurostimulant. Intense light scattering in those large brains makes them inaccessible by state-of-the-art two-photon or light-sheet/field microscopy methods, which are also currently incapable of capturing such large volumes in real time. Here FONT was found to provide time-resolved 3D reconstructions across these highly scattering brains (Figure 4). Five volumes of interest (VOIs) were selected for analysis of the dynamics of GCaMP5G using time series of both the optoacoustic (absorption) and the planar fluorescence signals during neural activation (Figure 4b and Supplementary Fig. S2; to better match the respective spatiotemporal dynamics the OA traces were averaged over (0.3 mm)^3^ volumes). According to the optoacoustic signal traces in Figure 4b, activation patterns associated with high-contrast calcium-related changes occurred mainly in deep-brain areas with maximal contrast ΔOA/OA_0_=8.5, whereas voxels close to the surface actually showed a slight decrease in activity (blue VOI). However, as compared with true 3D information provided by the tomographic optoacoustic reconstructions, whose spatial resolution is not affected by the intense light scattering, the planar fluorescence lacks optical sectioning and thus may result in wrong conclusions based on smeared subsurface information averaged over large volumes^28^. Only when activity appears mostly limited to superficial regions of the brain where light scattering is less significant, a relatively high correlation between the optoacoustic and epifluorescence readings was observed (Supplementary Fig. S2); see also the gray VOI placed over a superficial part of the optic nerve in Figure 4b and Supplementary Video 3, which shows a similar overall trend in both the fluorescence and optoacoustic modality, except for the generally faster fluctuations in the optoacoustic signals. On the other hand, signal variations detected by optoacoustics in the pink and yellow VOIs located deeper in the brain could not be well identified using planar fluorescence images. To better understand the *in situ* spatiotemporal characteristics of the OA signal, we defined short-line segment region of interest through single (unsmoothed) slices of the time-resolved 3D optoacoustic whole-brain data (Figure 4c and Supplementary Fig. S3) and analyzed their spatiotemporal profiles (Figure 4d). Using this analysis we observed signal transients in features located deep within the highly scattering brain of the adult zebrafish, whose temporal dynamics had a short characteristic rise time and whose dimensions were nearly resolution-limited (260 ms and 135 μm FWHM, respectively, in Figure 4e). The set of rapid spatiotemporal transients observed in the analyzed data set (*n*=6 optoacoustic image slices originating from two isolated brain preparations) had onset time constants ranging from 120 to 260 ms with resolvable image features in the range of 90–135 μm (further examples shown in Supplementary Fig. S3). Note that some of the unaveraged optoacoustic profiles exhibit negative signal values, which are attributed to secondary-image reconstruction artifacts caused by the partial tomographic coverage and limited detection bandwidth of the optoacoustic detection array^29^. Overall, the simultaneous dual-mode-imaging sessions demonstrated that epifluorescence fails to faithfully identify the calcium fluxes corresponding to deep-brain regions, which are readily resolvable by FONT with high spatiotemporal resolution.

The true strength and relative merits of the FONT approach can be appreciated when assessing its combined spatiotemporal-resolution performance corresponding to the respective fields of view. Indeed, state-of-the-art volumetric optical microscopy^5, 30, 31, 32^ has significantly better spatial resolution dictated by the optical diffraction limit, but the typical volumes that can be captured with millisecond-scale temporal resolution are orders of magnitude smaller as compared with FONT. Photon scattering remains the fundamental physical limitation for those methods; thus, the imaged volume cannot be extended beyond several 100 s of microns in the depth direction, rendering those methods incapable of large-scale recordings from entire scattering brains of adult fish or mice. On the opposite edge of the performance scale are macroscopic neuroimaging methods, such as functional magnetic resonance imaging^33, 34^ or optical diffusion techniques^35^. Those are suitable for visualizing large-brain volumes, but the spatiotemporal performance is inferior to FONT while the detected contrast is mainly limited to indirect representation of neuronal activity via slow hemodynamic responses.

Another issue that has to be carefully considered when evaluating the performance of new imaging modalities, such as our method, is scalability. For example, a cellular-scale spatial resolution in all three dimensions would be currently unrealistic to anticipate when imaging an entire mouse brain. This is because the number of voxels to be reconstructed in real time would be tremendously high in this case (~10^8^). Moreover, this type of performance would necessitate implementation of high-frequency ultrasound arrays with extreme density of elements that might not have sufficient detection sensitivity. Instead, matrix array probes with different spatial resolution and FOV could be used to image different brain sizes. For example, whereas FONT2 and FONT3 are suitable for imaging the zebrafish nervous system, the lower-resolution FONT1 system with FOV >1 cm^3^ can readily be used for real-time visualization of an entire brain of a live intact mouse using near-infrared wavelengths.

When considering imaging of neural activity in the mouse brain, one crucial aspect is the strongly limited penetration of light into highly vascularized mammalian tissues in the visible range. Light at the GCaMP excitation wavelength is strongly absorbed by blood, generating a strong optoacoustic signal background^16^. As a result, the currently existing calcium indicators are inadequate for deep-tissue optoacoustic imaging of the mouse brain because of the strong background absorption by blood and insufficient light penetration in the visible range. In addition, the vast majority of calcium indicators are optimized for fluorescence imaging, mainly exhibiting changes in their fluorescent quantum yield in response to calcium influx^36^ rather than their absorption cross-section, also making those indicators less practical for generating measurable optoacoustic signal variations. High-resolution imaging of the whole mouse brain is further challenged by acoustic distortions and attenuation of high-frequency ultrasound waves by the skull^37^. Nevertheless, our recent demonstration of *in vivo* imaging of cells labeled with near-infrared fluorescent proteins in deep mouse brain using multispectral optoacoustic tomography^38^ clearly suggests that development of analogous calcium-sensitive indicators in the near-infrared is expected to facilitate translation of the FONT technology into mammalian brains.

## Conclusions

In summary, we demonstrated a novel optoacoustic imaging platform for direct imaging of spatiotemporal neural activity across entire light-scattering brains while maintaining similar values of spatial resolution at highly scalable depths. Our study is also, to the best of our knowledge, the first to examine the optoacoustic signature of modern GECIs, showing that the strong changes in GCaMP5G fluorescence are directly related to their optoacoustic signature. As FONT uses a widely tunable nanosecond optical parametric oscillator laser technology, it can be conveniently tuned to work with a large array of other functional probes, including, for instance, the newer generation of GCaMP6 probes^13^ and red-shifted probes like RGECO^39^, or future sensors optimized for *in vivo* deep-tissue optoacoustic detection providing high extinction coefficient changes in the near-infrared window as well as lower quantum yield. Furthermore, the ability of FONT to simultaneously track movements and neural activation in three dimensions from living unrestrained organisms could form a basis for behavioral studies not currently possible with other optical neuroimaging techniques owing to their limited fields of view. Whereas the current spatial resolution still does not allow to distinguish individual cells, future generations of the described tomographic approach could potentially utilize higher-frequency transducer technology and/or super-resolution strategies for fast functional observations at the cellular scale. However, FONT's high temporal resolution is important even without the ability to resolve individual cells, as neural population calcium dynamics imaged with low-resolution systems still clearly convey very rapid timescale information^40, 41^ (see also Figure 3). In addition, the high sensitivity of optoacoustics to a variety of intrinsic absorption tissue contrasts, most prominently the oxy- and de-oxy hemoglobin, is well established^42, 43^. This may provide highly complementary information to the functional calcium imaging and thus enhance the amount of spectrally- and time-resolved volumetric information available for the five-dimensional optoacoustic studies looking at coupling between the vascular changes and nervous system in more complex animal models. By virtue of combining the contrast abilities of both microscopic and macroscopic functional neuroimaging methods with its unprecedented spatiotemporal resolution performance, FONT fills an important performance gap in the current neuroimaging technology and opens new prospects for large-scale observations of neural networks.

## Author contributions

DR, SS and GGW proposed and designed the project. DR and XLDB designed the optoacoustic imaging system. AL and GGW provided the animal models. XLDB, GS, AL and MK carried out the experiments. GS and XLDB implemented image reconstruction and processing algorithms and analyzed the data. DR, SS, GGW and VN supervised the study. All authors discussed the results and contributed to writing the manuscript.

## Supplementary Material

Supplementary Informatin
