# Supplementary material for: Functional optoacoustic neuro-tomography for scalable whole-brain monitoring of calcium indicators
Source: Light Sci Appl. 2016 Dec 2;5(12):e16201–. doi: 10.1038/lsa.2016.201 (PMC6059886; doi:10.1038/lsa.2016.201)
Supplement: Supplementary Informatin [file lsa2016201x1.pdf]

Supplementary Information for "Functional optoacoustic neuro-tomography (FONT) for scalable whole-brain monitoring of calcium indicators"

X. Luís Deán-Ben[1,*], Gali Sela[1,*], Antonella Lauri[1,2,*], Moritz Kneipp[1,3], Vasilis Ntziachristos[1,3], Gil G. Westmeyer[1,2,3,†], Shy Shoham[4,†], and Daniel Razansky[1,3,†]

[1]*Institute for Biological and Medical Imaging (IBMI), Helmholtz Center Munich, Neuherberg, Germany*

[2]*Institute of Developmental Genetics, Helmholtz Center Munich, Neuherberg, Germany*

[3]*Department of Medicine, Technical University of Munich, Germany*

[4]*Department of Biomedical Engineering, Technion – Israel Institute of Technology, Haifa, Israel*

* Equal contribution

† Correspondence to S. S (sshoham@bm.technion.ac.il), G. G. W. (gil.westmeyer@tum.de) or D. R. (dr@tum.de)

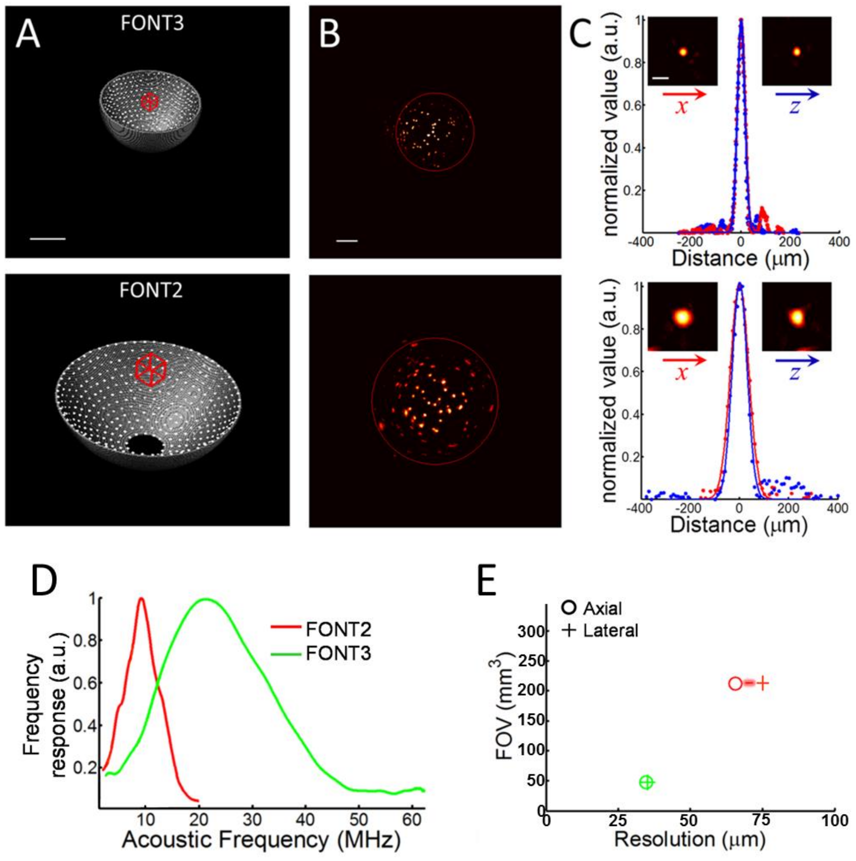

Supplementary Figure 1. Characterization of the FONT systems. (**A**) Geometry of the two matrix detection arrays introduced in this work showing distribution of the ultrasound detection elements along the spherical detection surfaces. The effective imaging field of view for each array is indicated by the red box. Scale bar – 10mm. (**B**) The field of view was characterized by imaging sparsely distributed absorbing microspheres having diameter of 50 μm (FONT2) or 20μm (FONT3). Scale bar in the reconstructed optoacoustic images is 1mm. (**C**) Optoacoustic signal profiles from individual spheres located approximately in the center of the detection array. Profiles along both the axial and lateral directions are shown. The corresponding optoacoustic images are shown in the insets (scale bar - 100μm). (**D**) Frequency response of FONT2 (red) and FONT3 (green). (**E**) Characterization of the field of view versus spatial resolution, which was performed by imaging absorbing microspheres with diameters between 20 to 50μm.

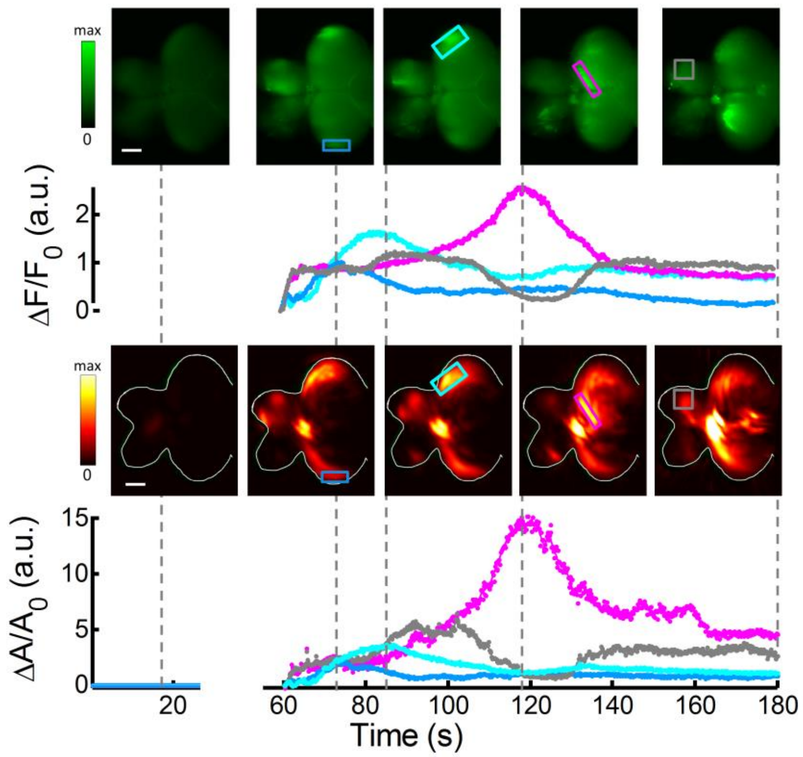

Supplementary Figure 2. Activity monitored in shallow areas of an isolated scattering brain. Higher correlation between the optoacoustic and epi-fluorescence measurements was observed in case the activation mainly occurred in shallow areas of a highly scattering adult zebrafish brain (presumably due to a non-uniform distribution of the neurostimulating agent). Traces of the fluorescence (top) and optoacoustic (bottom) signal changes in four different areas located at shallow depths (all signal changes are normalized to the resting signal levels). Note that the optoacoustic images were averaged here over volumes of $(0.3\ \text{mm})^3$ and are shown as maximal intensity projections along the *z* axis. Colors of the graphs correspond to the labeling of the corresponding areas in the insets. Snapshots acquired at 5 different time points before and after administration of the neurostimulant are shown (scale bar - 500 µm). The administration phase caused image artifacts and is therefore excluded from the graphs.

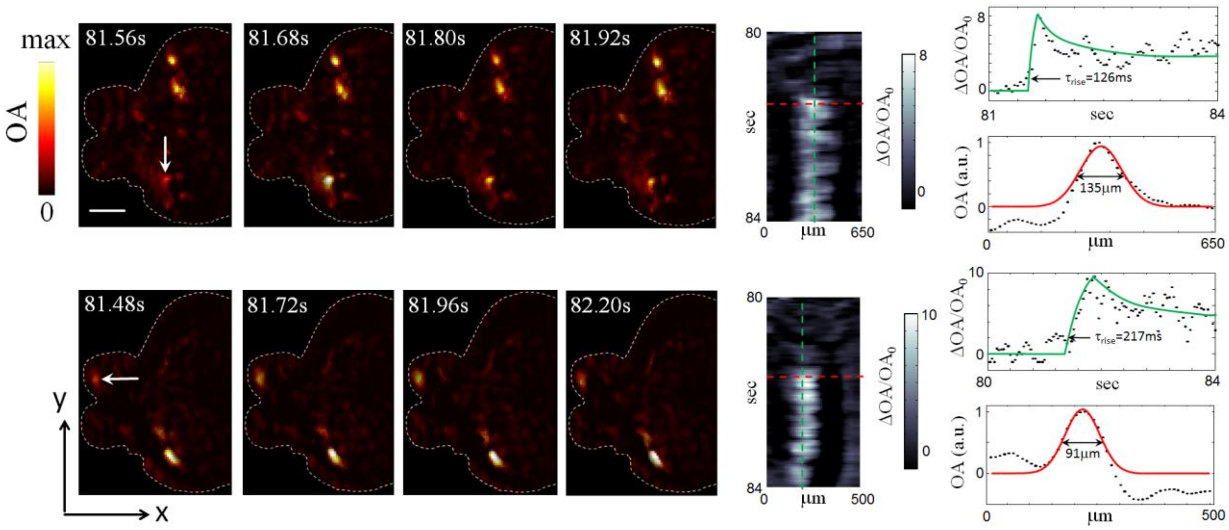

Supplementary Figure 3. Additional examples of the observed spatio-temporal resolution analysis in single un-averaged slices through the time-resolved 3D optoacoustic data. The slices were randomly selected from the adult zebrafish time-lapse optoacoustic data and the analysis was performed as described in the captions of Fig. 4 (panels C and D).

Supplementary Videos

Supplementary Video 1. Volumetric optoacoustic images of 6dpf freely swimming wild type larvae acquired at 750 nm illumination wavelength recorded at 100 frames per second, the maximal volumetric imaging speed of the system. Note that the video was compiled by skipping between relatively informative time points using a crossfade effect.

Supplementary Video 2. Immobilized larvae showing a neural response as a result of introduction of a neurostimulating agent. Similar responses are evident in fluorescence (top) and optoacoustic (absorption - bottom) videos, both recorded at 25 frames per second. Two areas are marked by blue and red rectangles on the fluorescence videos and the traces of the fluorescence and absorption changes in these areas are given on the right. (scale bar 500µm).

Supplementary Video 3. Post stimulation activation in isolated brain of an adult fish, as recorded in both fluorescence (top left panel) and optoacoustic (top right panel) modes at 25 frames per second. The 3D optoacoustic reconstruction is shown on the bottom panel. The background (pre-activation) optoacoustic contrast of the brain is visualized in grey color scheme while the differential signals post activation are depicted in hot color scheme. All color schemes are normalized to different values and thus correspond to arbitrary units (scale bar 500µm). The same dataset is also analyzed in Fig. 4.
